# Acute Pesticide Illnesses Associated with Off-Target Pesticide Drift from Agricultural Applications: 11 States, 1998–2006

**DOI:** 10.1289/ehp.1002843

**Published:** 2011-06-06

**Authors:** Soo-Jeong Lee, Louise Mehler, John Beckman, Brienne Diebolt-Brown, Joanne Prado, Michelle Lackovic, Justin Waltz, Prakash Mulay, Abby Schwartz, Yvette Mitchell, Stephanie Moraga-McHaley, Rita Gergely, Geoffrey M. Calvert

**Affiliations:** 1 Division of Surveillance, Hazard Evaluations, and Field Studies, National Institute for Occupational Safety and Health, Centers for Disease Control and Prevention, Cincinnati, Ohio, USA; 2 California Environmental Protection Agency, Sacramento, California, USA; 3 Public Health Institute, Oakland, California, USA; 4 Texas Department of State Health Services, Austin, Texas, USA; 5 Washington State Department of Health, Olympia, Washington, USA; 6 Louisiana Department of Health and Hospitals, New Orleans, Louisiana, USA; 7 Oregon Health Authority, Portland, Oregon, USA; 8 Florida Department of Health, Tallahassee, Florida, USA; 9 Michigan Department of Community Health, Lansing, Michigan, USA; 10 New York State Department of Health, Troy, New York, USA; 11 New Mexico Department of Health, Albuquerque, New Mexico, USA; 12 Iowa Department of Public Health, Des Moines, Iowa, USA

**Keywords:** agriculture, drift, pesticides, poisoning, surveillance

## Abstract

Background: Pesticides are widely used in agriculture, and off-target pesticide drift exposes workers and the public to harmful chemicals.

Objective: We estimated the incidence of acute illnesses from pesticide drift from outdoor agricultural applications and characterized drift exposure and illnesses.

Methods: Data were obtained from the National Institute for Occupational Safety and Health’s Sentinel Event Notification System for Occupational Risks–Pesticides program and the California Department of Pesticide Regulation. Drift included off-target movement of pesticide spray, volatiles, and contaminated dust. Acute illness cases were characterized by demographics, pesticide and application variables, health effects, and contributing factors.

Results: From 1998 through 2006, we identified 2,945 cases associated with agricultural pesticide drift from 11 states. Our findings indicate that 47% were exposed at work, 92% experienced low-severity illness, and 14% were children (< 15 years). The annual incidence ranged from 1.39 to 5.32 per million persons over the 9-year period. The overall incidence (in million person-years) was 114.3 for agricultural workers, 0.79 for other workers, 1.56 for nonoccupational cases, and 42.2 for residents in five agriculture-intensive counties in California. Soil applications with fumigants were responsible for the largest percentage (45%) of cases. Aerial applications accounted for 24% of cases. Common factors contributing to drift cases included weather conditions, improper seal of the fumigation site, and applicator carelessness near nontarget areas.

Conclusions: Agricultural workers and residents in agricultural regions had the highest rate of pesticide poisoning from drift exposure, and soil fumigations were a major hazard, causing large drift incidents. Our findings highlight areas where interventions to reduce off-target drift could be focused.

Pesticide drift, which is the off-target movement of pesticides, is recognized as a major cause of pesticide exposure affecting people as well as wildlife and the environment. In the United States in 2004, > 1,700 investigations were conducted in 40 states because of drift complaints, and 71% of the incident investigations confirmed that drift arose from pesticide applications to agricultural crops (Association of American Pesticide Control Officials 2005). Pesticide drift has been reported to account for 37–68% of pesticide illnesses among U.S. agricultural workers [California Department of Pesticide Regulation (CDPR) 2008; Calvert et al. 2008]. Community residents, particularly in agricultural areas, are also at risk of exposure to pesticide drift from nearby fields. Agricultural pesticides are often detected in rural homes (Harnly et al. 2009; Quandt et al. 2004). Alarcon et al. (2005) reported that 31% of acute pesticide illnesses that occurred at U.S. schools were attributed to drift exposure.

The occurrence and extent of pesticide drift are affected by many factors, such as the nature of the pesticide (e.g., fumigants are highly volatile, which increases their propensity for off-site movement [U.S. Environmental Protection Agency (U.S. EPA) 2010], equipment and application techniques (e.g., size and height of the spray nozzles), the amount of pesticides applied, weather (e.g., wind speed, temperature inversion), and operator care (Hofman and Solseng 2001). Pesticide applicators are required to use necessary preventive measures and to comply with label requirements to minimize pesticide drift. Pesticide regulations such as the Federal Insecticide, Fungicide, and Rodenticide Act (FIFRA) and EPA’s Worker Protection Standard require safety measures for minimizing the risk of pesticide exposure (U.S. EPA 2008, 2009), and many states have additional regulations for drift mitigation (Feitshans 1999).

Better understanding about the magnitude, trend, and characteristics of pesticide poisoning from drift exposure of agricultural pesticides would assist regulatory authorities with regulatory, enforcement, and education efforts. The purpose of this study was to estimate the magnitude and incidence of acute pesticide poisoning associated with pesticide drift from outdoor agricultural applications in the United States during 1998–2006 and to describe the exposure and illness characteristics of pesticide poisoning cases arising from off-target drift. We also examined factors associated with illness severity and large events that involved five or more cases.

## Materials and Methods

Data on acute pesticide poisoning cases were obtained from the National Institute for Occupational Safety and Health (NIOSH)’s Sentinel Event Notification System for Occupational Risks (SENSOR)-Pesticides program and CDPR’s Pesticide Illness Surveillance Program (PISP). The SENSOR-Pesticides program has collected pesticide poisoning surveillance data from 12 states using standardized definitions and variables available since 1998 (Calvert et al. 2010). This study included data from 11 states for the following years: Arizona, 1998–2000; California, 1998–2006; Florida, 1998–2006; Iowa, 2006; Louisiana, 2000–2006; Michigan, 2000–2006; New Mexico, 2005–2006; New York, 1998–2006; Oregon, 1998–2006; Texas, 1998–2006; and Washington, 2001–2006. North Carolina, which joined SENSOR-Pesticides in 2007, was not included. Because each state removes personal identifiers from the data before submission to the Centers for Disease Control and Prevention (CDC), this study was exempt from consideration by the federal Human Subjects Review Board.

Participating surveillance programs identify cases from multiple sources, including health care providers, poison control centers, workers’ compensation claims, and state or local government agencies. They collect information on the pesticide exposure incident through investigation, interview, and medical record review. In California, on some occasions, such as large drift events, active surveillance is undertaken for further case finding by interviewing individuals living or working within the vicinity affected by the off-target drift (Barry et al. 2010). Although the SENSOR-Pesticides program focuses primarily on occupational pesticide poisoning surveillance, all of the SENSOR-Pesticides state programs except California collect data on both occupational and nonoccupational cases. In California, PISP captures both occupational and nonoccupational cases. SENSOR-Pesticides and PISP classify cases based on the strength of evidence for pesticide exposure, health effects, and the known toxicology of the pesticide and use slightly different criteria for case classification categories (Calvert et al. 2010). This study restricted the analyses to cases classified as definite, probable, possible, or suspicious by SENSOR-Pesticides and definite, probable, or possible by PISP. We also performed analyses restricted to definite and probable cases only. Because the findings from these restricted analyses were similar to those that included all four classification categories (i.e., definite, probable, possible, or suspicious), only the findings that used the four classification categories are reported here.

In this study, a drift case was defined as acute health effects in a person exposed to pesticide drift from an outdoor agricultural application. Drift exposure included any of the following pesticide exposures outside their intended area of application: *a*) spray, mist, fumes, or odor during application; *b*) volatilization, odor from a previously treated field, or migration of contaminated dust; and *c*) residue left by offsite movement. Our drift definition is broader than U.S. EPA’s “spray or dust drift” definition, which excludes postapplication drift caused by erosion, migration, volatility, or windblown soil particles (U.S. EPA 2001). A drift event was defined as an incident where one or more drift cases experienced drift exposure from a particular source. Both occupational and nonoccupational cases were included. An occupational case was defined as an individual exposed while at work. Among occupational cases, agricultural workers were identified using 1990 and 2002 Census Industry Codes (CICs): 1990 CICs, 010, 011, 030; 2002 CICs, 0170, 0180, 0290 (U.S. Census Bureau 1992, 2005).

**Figure 1:**
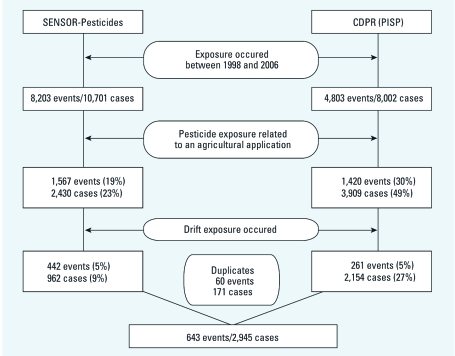
Eligible pesticide drift events and cases, 11 states, 1998–2006.

Figure 1 presents the process of case selection. We selected cases if exposed to pesticides applied for agricultural use including farm, nursery, or animal production, and excluded cases exposed by ingestion, direct spray, spill, or other direct exposure. We then manually reviewed all case reports and excluded persons exposed to pesticides used for indoor applications (e.g., greenhouses, produce packing facilities), persons exposed within a treated area (e.g., pesticide applicators exposed by pesticides blown back by wind, workers working within or passing through the field being treated), and persons exposed to pesticides being mixed, loaded, or transported. Drift cases therefore represented the remaining 9% and 27% of all pesticide illness cases identified by the SENSOR-Pesticides and PISP, respectively. We also searched for duplicates from the two programs identifying California cases. Because personal identifiers were unavailable, date of exposure, age, sex, active ingredients, and county were used for comparison. A total of 60 events and 171 cases were identified by both California programs. These were counted only once and were included only in the PISP total.

Drift events and cases were analyzed by the following variables: state, year, and month of exposure, age, sex, location of exposure, health effects, illness severity, pesticide functional and chemical class, active ingredient, target of application, application equipment, detection of violations, and factors contributing to the drift incident. U.S. EPA toxicity categories ranging from toxicity I (the most toxic) to IV (the least toxic) were assigned to each product (U.S. EPA 2007). Cases exposed to multiple products were assigned to the toxicity category of the most toxic pesticide they were exposed to. Illness severity was categorized into low, moderate, and high using criteria developed by the SENSOR-Pesticides program (Calvert et al. 2010). Low severity refers to mild illnesses that generally resolve without treatment. Moderate severity refers to illnesses that are usually systemic and require medical treatment. High severity refers to life-threatening or serious health effects that may result in permanent impairment or disability. Contributing factors were retrospectively coded with available narrative descriptions. One NIOSH researcher (S.J.L.) initially coded contributing factors for all cases. Next, for SENSOR-Pesticides cases, state health department staff reviewed the codes and edited them as necessary. Any discrepancies were resolved by a second NIOSH researcher (G.M.C.). For PISP cases, relatively detailed narrative descriptions were available for all incidents. These narratives summarize investigation reports provided by county agriculture commissioners, who investigate all suspected pesticide poisoning cases reported in their county. After initial coding, the two NIOSH researchers discussed those narratives that lacked clarity to reach consensus.

*Data analysis.* Data analysis was performed with SAS software (version 9.1; SAS Institute Inc., Cary, NC). Descriptive statistics were used to characterize drift events and cases. Incidence rates were calculated by geographic region, year, sex, and age group. The numerator represented the total number of respective cases in 1998–2006. Denominators were generated using the Current Population Survey microdata files for the relevant years (U.S. Census Bureau 2009). For total and nonoccupational rates, the denominators were calculated by summing the annual average population estimates. A nonoccupational rate for agriculture-intensive areas was calculated by selecting the five counties in California where the largest amounts of pesticides were applied in 2008 (Fresno, Kern, Madera, Monterey, and Tulare) (CDPR 2010). For occupational rates, the denominators were calculated by summing the annual employment estimates including both “employed at work” and “employed but absent.” The denominator for agricultural workers was obtained using the same 1990 and 2002 CICs used to define agricultural worker cases (U.S. Census Bureau 1992, 2005). Moreover, in California, where data on pesticide use are available, incidence was calculated per number of agricultural applications and amount of pesticide active ingredient applied (CDPR 2009). Incidence trend over time was examined by fitting a Poisson regression model of rate on year and deriving the regression coefficient and its 95% confidence interval (CI).

Drift events were dichotomized by the size of events into small events involving < 5 cases and large events involving ≥ 5 cases. This cut-point was based on one of the criteria used by the CDPR to prioritize event investigations (CDPR 2001). Illness severity was dichotomized as low and moderate/high. Simple and multivariable logistic regressions were performed. Odds ratios (ORs) and 95% CIs were calculated.

## Results

*Number and incidence of drift events and cases.* From 1998 through 2006, we identified 643 events and 2,945 illness cases associated with pesticide drift from agricultural applications (Figure 1). Of these, 382 events (59%) and 791 cases (27%) were identified by SENSOR-Pesticides (excluding 60 events and 171 cases also identified by PISP), and 261 events (41%) and 2,154 cases (73%) were identified by PISP. Drift cases consisted of 53 definite (1.8%), 2,019 probable (68.6%), 823 possible (27.9%), and 50 suspicious (1.7%) cases. Among drift cases, 1,565 (53%) were nonoccupational and 1,380 (47%) were occupational. Agricultural workers accounted for 73% (*n* = 1,010) of the occupational cases. A total of 340 events (53%) occurred between May and August, and these involved 1,407 cases (48%).

The overall incidence rate of drift-related pesticide poisoning was 2.93 per million person-years (Table 1). The rates of nonoccupational and occupational drift-related pesticide poisoning were 1.56 and 2.89 per million persons-years, respectively. Among occupational cases, the rate was 114.3 for agricultural workers and 0.79 for all other workers. Among nonoccupational cases identified in California, the rate was 42.2 for residents in the five agriculture-intensive counties and 0.61 for residents of all other California counties (data not shown). The rate was highest in the western states for both nonoccupational and occupational cases (Table 1). In California, per 100,000 agricultural applications, 1.6 drift events and 11.8 cases were identified; per 10 million pounds applied, 1.9 events and 14.4 cases were identified (data not shown).

**Table 1:** Number and incidence rate*a* of off-target drift events and pesticide poisoning cases by year, region, sex, and age, 11 states, 1998–2006.

Drift cases																						
Nonoccupational cases			Occupational cases												
			All cases					Agricultural worker cases					Other worker cases				
Drift events		Population estimate*b*	Employment estimate*b*^,d^	Employment estimate*b*	Total rate
Variable	Count (%)		Count	Rate	Count	Rate*c*	Count	Rate	Count	Rate
Total		643	(100)		2,945		1,004.1		2.93		1,565		1.56		1,010		8.83		114.33		370		468.0		0.79		2.89
Year of exposure (no. states included)																											
1998 (6)		60	(9.3)		130		93.6		1.39		46		0.49		45		1.11		40.46		39		43.2		0.90		1.90
1999 (6)		82	(12.8)		407		95.0		4.28		273		2.87		72		1.12		64.22		62		44.1		1.41		2.97
2000 (8)		64	(10.0)		193		110.3		1.75		76		0.69		93		1.24		74.94		24		51.8		0.46		2.21
2001 (8)		88	(13.7)		177		112.6		1.57		98		0.87		43		1.12		38.47		36		52.5		0.69		1.47
2002 (8)		81	(12.6)		580		113.7		5.10		271		2.38		281		1.11		252.33		28		52.2		0.54		5.80
2003 (8)		75	(11.7)		348		116.4		2.99		265		2.28		43		0.79		54.64		40		53.7		0.74		1.52
2004 (8)		47	(7.3)		232		117.4		1.98		43		0.37		177		0.75		235.33		12		54.7		0.22		3.41
2005 (9)		70	(10.9)		642		120.6		5.32		409		3.39		168		0.75		224.77		65		56.8		1.14		4.05
2006 (10)		76	(11.8)		236		124.5		1.90		84		0.67		88		0.84		104.53		64		59.1		1.08		2.54
Region																											
West*e*		433	(67.3)		2,484		397.9		6.24		1,240		3.12		933		4.44		210.20		311		184.9		1.68		6.57
South*f*		193	(30.0)		426		365.6		1.17		311		0.85		59		3.25		18.17		56		170.7		0.33		0.66
East/central*g*		17	(2.6)		35		240.6		0.15		14		0.06		18		1.15		15.68		3		112.5		0.03		0.18
Sex		NA																					0.0				
Male					1,560		491.6		3.17		742		1.51		554		6.90		80.27		264		251.6		1.05		3.16
Female					1,360		512.5		2.65		807		1.57		448		1.93		231.90		105		216.5		0.49		2.53
Unknown					25		—		—		16		—		8		—		—		1		—		—		—
Age (years)		NA																									
< 15					418		221.2		1.89		415		1.88		3		—		—		0		—		—		—
15–24					398		142.0		2.80		182		1.28		182		1.44		126.39		34		67.8		0.50		3.12
25–34					453		140.0		3.24		140		1.00		240		1.81		132.53		73		106.8		0.68		2.88
35–44					458		156.7		2.92		181		1.16		187		2.08		89.89		90		122.3		0.74		2.23
45–54					306		136.1		2.25		172		1.26		78		1.59		49.00		56		104.6		0.54		1.26
55–64					164		90.9		1.80		103		1.13		37		1.10		33.61		24		52.0		0.46		1.15
≥ 65					92		117.2		0.78		80		0.68		9		0.81		11.11		3		14.6		0.21		0.78
Unknown					656		—		—		292		—		274		—		—		90		—		—		—
Abbreviations: —, the denominator was not available and thus a rate was not calculated, NA, for sex and age, counting the number of events was not applicable. **a**Per 1,000,000 persons. **b**Cases and employment estimates of agricultural workers were defined with 1990 and 2002 CICs (010, 011, 030 and 0170, 0180, 0290, respectively). **c**Numbers (in millions) were estimated using the Current Population Survey data (U.S. Census Bureau 2009). Participating years vary by state; only years of participation were included. **d**Denominators were population estimates. **e**Arizona, California, New Mexico, Oregon, Washington. **f**Florida, Louisiana, Texas. **g**Iowa, Michigan, New York.																											

**Figure 2:**
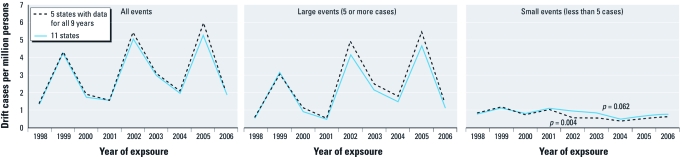
Incidence rate of pesticide poisoning associated with off-target drift exposure over time, 11 states, 1998–2006.

The total annual incidence rate ranged from 1.39 to 5.32 per million persons over the 9-year time period (Table 1). Over time, the rate of drift cases involved in large events showed the same pattern as the rate of all drift cases, showing a spike every 3 years (Figure 2). The rate of drift cases involved in small events varied within a narrow range from 0.49 to 1.11, and we found no significant rate change over this time period; however, for the five states that provided data for all 9 years, we found a significant decrease in the rate (i.e., an estimated 9% decrease per year; 95% CI, 3–15%; *p* = 0.004).

Men comprised 53% of all cases (Table 1). The rate by sex was similar among nonoccupational cases. For occupational cases, the rate was 1.25 times higher in male workers than in female workers but 2.89 times higher in female agricultural workers than in male agricultural workers. Among nonoccupational cases, children < 15 years of age accounted for 33% of cases with known age and showed the highest rate (1.88/million person-years; Table 1).

*Responsible pesticides, application targets, and application equipment.* In 430 (67%) of 643 drift events, exposure was to pesticides from a single functional class (Table 2). Insecticides were the most commonly identified (31% of events), accounting for 23% (*n* = 678) of all cases. Fumigants were involved in only 8% of drift events but accounted for 45% (*n* = 1,330) of all cases. Organophosphorus compounds were the most common pesticide chemical class involved in drift events (28%). Most cases (66%) were exposed to toxicity I (high toxicity) pesticides.

**Table 2:** Off-target drift events and pesticide poisoning cases by pesticide and application characteristics, 11 states, 1998–2006.

Drift events (*n* = 643)		Drift cases					
		Total (*n* = 2,945)		Occupational *n* = 1,380 (%)	Nonoccupational *n* = 1,565 (%)
Variable	*n* (%)		*n* (%)		
Pesticide functional class										
Insecticide only		198	(30.8)		678	(23.0)		32.9		14.3
Herbicide only		108	(16.8)		195	(6.6)		4.0		8.9
Fungicide only		29	(4.5)		64	(2.2)		3.7		0.8
Fumigant only		52	(8.1)		1,330	(45.2)		27.0		61.2
Other, single		43	(6.7)		87	(3.0)		2.8		3.1
Multiple		207	(32.2)		585	(19.9)		29.4		11.4
Unknown		6	(0.9)		6	(0.2)		0.2		0.2
Common pesticide chemical class*a*										
Organophosphorus compound		181	(28.1)		660	(22.4)		36.7		9.8
Inorganic compound		87	(13.5)		231	(7.8)		11.1		5.0
Pyrethroid		52	(8.1)		207	(7.0)		9.6		4.7
Dithiocarbamates*b*		47	(7.3)		726	(24.7)		22.5		26.5
*N*-Methyl carbamates		33	(5.1)		71	(2.4)		4.1		1.0
Chlorophenoxy compound		26	(4.0)		47	(1.6)		0.9		2.2
Triazines		11	(1.7)		34	(1.2)		1.1		1.2
Maximum toxicity category										
I		203	(31.6)		1,944	(66.0)		59.9		71.4
II		167	(26.0)		468	(15.9)		21.2		11.2
III		154	(24.0)		327	(11.1)		13.6		8.9
Unknown		119	(18.5)		206	(7.0)		5.2		8.6
Application target										
Fruit crops		189	(29.4)		588	(20.0)		27.6		13.2
Grain/fiber/grass crops		185	(28.8)		411	(14.0)		12.8		15.0
Vegetable crops		85	(13.2)		374	(12.7)		22.9		3.7
Soil		55	(8.6)		1,337	(45.4)		27.5		61.2
Landscape/forest		32	(5.0)		64	(2.2)		2.8		1.7
Undesired plants		29	(4.5)		44	(1.5)		0.9		2.0
Other (e.g., miscellaneous crops, seed, livestock farm)		27	(4.2)		66	(2.2)		2.0		2.5
Unknown		41	(6.4)		61	(2.1)		3.6		0.8
Application equipment										
Aerial applicator		249	(38.7)		695	(23.6)		32.0		16.2
Handheld or backpack sprayer		24	(3.7)		63	(2.1)		3.8		0.6
Chemigation		22	(3.4)		752	(25.5)		16.4		33.5
Soil injector		20	(3.1)		558	(18.9)		10.0		26.8
Other ground applicator		254	(39.5)		747	(25.4)		32.6		19.0
Multiple		8	(1.2)		41	(1.4)		0.2		2.4
Unknown		66	(10.3)		89	(3.0)		4.9		1.4
**a**Categories with the largest numbers of cases. Events and cases can be exposed to multiple categories. **b**Mostly from single products.										

For the intended application targets, 71% of events involved applications to fruit, grain/fiber/grass, or vegetable crops (Table 2). Soil applications accounted for 9% of drift events and 45% of all cases. For application equipment, aerial applications (e.g., by airplane) were responsible for 39% of drift events, accounting for 24% of all cases. Chemigation (i.e., application via an irrigation system) or soil injectors were used in 7% of drift events and accounted for 44% of cases. All soil injector events and 95% of chemigation events involved the use of fumigants applied to soil (data not shown).

*Location of exposure and health effects.* Common exposure locations were private residences (44%) and farms/nurseries (37%; Table 3). More than half of cases experienced ocular (58%) or neurological (53%) symptoms or signs, and illness severity was low for most cases (92%; Table 3). Moderate/high severity illness was significantly associated with females, older age groups, and exposure to multiple active ingredients, before and after controlling for other case and pesticide characteristics (*p* < 0.05; Table 4). Compared with fumigants, exposures to herbicides, insecticides, or multiple classes were significantly associated with moderate/high illness. Table 5 lists 15 active ingredients most commonly found among drift cases and their distribution according to illness severity.

**Table 3:** Location of exposure, health effects, and illness severity of drift cases (*n* = 2,945).

Variable	Percent
Location of exposure	
Private residence	44.5
Farm/nursery	36.7
Road/right-of-way	5.6
School	3.6
Agricultural processing facility	2.4
Other/unknown	7.2
Health effect*a*	
Eye (e.g., pain/irritation/inflammation, lacrimation)	58.2
Neurological (e.g., headache, paresthesia, dizziness)	52.8
Respiratory (e.g., dyspnea, respiratory tract pain/irritation, cough)	47.8
Gastrointestinal (e.g., vomiting, nausea, diarrhea, abdominal pain)	41.5
Skin (e.g., pruritus, pain/irritation, rash)	14.7
Cardiovascular (e.g., chest pain)	5.1
Other (e.g., fatigue, fever)	11.4
Illness severity	
Low	92.2
Moderate	7.3
High	0.5
**a**Cases may have been included in multiple categories.	

**Table 4:** Illness severity by case and pesticide characteristics.

Moderate/high severity (*n* = 230)		Low severity (*n* = 2,715)		Moderate/high severity (vs. low)		
Variable	*n* (%)		*n* (%)		OR (95% CI)	Adjusted OR*a* (95% CI)
Sex*b*										
Female		126	(54.8)		1,234	(45.5)		1.43 (1.09–1.87)		1.53 (1.15–2.04)
Male		104	(45.2)		1,456	(53.6)		Reference		Reference
Age (years)										
< 15		16	(7.0)		402	(14.8)		Reference		Reference
15–24		28	(12.2)		370	(13.6)		1.90 (1.01–3.57)		1.34 (0.68–2.62)
25–34		48	(20.9)		405	(14.9)		2.98 (1.66–5.33)		1.95 (1.02–3.71)
35–44		48	(20.9)		410	(15.1)		2.94 (1.64–5.27)		1.91 (1.02–3.58)
45–54		38	(16.5)		268	(9.9)		3.56 (1.95–6.52)		2.34 (1.24–4.41)
55–64		21	(9.1)		143	(5.3)		3.69 (1.87–7.27)		2.42 (1.20–4.91)
≥ 65		16	(7.0)		76	(2.8)		5.29 (2.54–11.03)		3.67 (1.72–7.86)
Unknown		15	(6.5)		641	(23.6)		0.59 (0.29–1.20)		0.63 (0.30–1.33)
Work related										
Yes		126	(54.8)		1,254	(46.2)		1.41 (1.08–1.85)		0.99 (0.70–1.40)
No/unknown		104	(45.2)		1,461	(53.8)		Reference		Reference
No. active ingredients										
1		90	(39.1)		1,719	(63.3)		Reference		Reference
> 1		140	(60.9)		996	(36.7)		2.72 (2.07–3.58)		1.42 (1.02–1.99)
Pesticide functional class										
Fumigant		35	(15.2)		1,295	(47.7)		Reference		Reference
Herbicides		33	(14.3)		162	(6.0)		7.54 (4.56–12.46)		4.10 (2.34–7.19)
Insecticide		79	(34.3)		599	(22.1)		4.88 (3.24–7.35)		3.34 (2.10–5.32)
Fungicides		2	(0.9)		62	(2.3)		1.19 (0.28–5.08)		0.77 (0.18–3.37)
Multiple		71	(30.9)		514	(18.9)		5.11 (3.37–7.76)		3.09 (1.85–5.16)
Other/unknown		10	(4.3)		83	(3.1)		4.46 (2.13–9.32)		2.82 (1.29–6.15)
**a**Adjusted for all other variables. **b**Excluded unknown cases.										

**Table 5:** Fifteen most common active ingredients for drift cases and percentage of moderate/high severity.

Cases exposed to single active ingredient		
Active ingredient	Functional class	Chemical class	Cases*a* (*n* = 2,945)	Total (*n* = 1,809)	Percent moderate/high severity (*n* = 90)*b*
Metam-sodium		Fumigant		Dithicarbamate		664		664		3
Chloropicrin		Fumigant		Trichloronitromethane		637		532		1
Chlorpyrifos		Insecticide		Organophosphate		240		49		10
Sulfur		Insecticide/fungicide		Inorganic compound		147		32		25
Mancozeb		Fungicide		Dithicarbamate		144		4		0
Methamidophos		Insecticide		Organophosphate		133		0		0
Malathion		Insecticide		Organophosphate		122		96		11
Spinosad		Insecticide		Spinosyn		107		1		0
Methyl bromide		Fumigant		Alkyl bromide		84		11		27
Dimethoate		Insecticide		Organophosphate		68		10		20
Cyfluthrin		Insecticide		Pyrethroid		59		2		0
Methomyl		Insecticide		*N*-Methyl carbamate		56		13		15
Atrazine		Herbicide		Triazine		54		8		0
λ-Cyhalothrin		Insecticide		Pyrethroid		52		39		3
Propargite		Acaricide/miticide		Sulfite ester		52		10		30
**a**Can be exposed to other active ingredients also. **b**High, *n* = 7; moderate, *n* = 83.										

*Size of drift events.* Most drift events involved a single case (*n* = 387, 60%). For multiperson events, 168 events (26% of the total) involved 2–4 cases, 78 events (12%) involved 5–29 cases, and 10 events (1.5%) involved ≥ 30 cases. Table 6 provides details on the 10 largest events. Detailed investigation reports of some of these events are available elsewhere (Barry et al. 2010; CDC 2004; O’Malley et al. 2005). The occurrence of large versus small events (events with ≥ 5 vs. < 5 cases) was significantly associated with the use of fumigants (compared with insecticides) and applications to soil, small fruit crops, or leafy vegetable crops (compared with other targets; *p* < 0.05; Table 7).

**Table 6:** Ten largest drift events, 1998–2006.

Cases					Pesticide application				
State	Year	Total (*n* = 1,293)	Occupational (*n* = 452)	Nonoccupational (*n* = 841)	Target	Equipment	Active ingredient
California		1999		170		6		164		Soil		Chemigation		Metam-sodium
California		2000		33		33		0		Almonds		Aerial application		Chlorpyrifos, propargite
California		2002		250		72		178		Soil		Soil injector		Metam-sodium
California		2002		123		123		0		Soil		Chemigation		Metam-sodium
California		2003		161		10		151		Soil		Soil injector		Chloropicrin
California		2004		122		122		0		Potatoes		Aerial application		Methamidophos
California		2005		324		1		323		Soil		Chemigation		Chloropicrin
California		2005		42		42		0		Soil		Chemigation		Metam-sodium
California		2005		34		34		0		Oranges		Ground sprayer		Cyfluthrin, spinosad
Texas		2005		34		9		25		Cotton		Ground sprayer		λ-Cyhalothrin

**Table 7:** Factors associated with large drift events (≥ 5 cases).

Small event (*n* = 555)		Large event (*n* = 88)		Large event (vs. small), OR (95% CI)
Factor	*n* (%)		*n* (%)	
Pesticide functional class								
Insecticide		172	(31.0)		26	(29.5)		Reference
Fumigant		29	(5.2)		23	(26.1)		5.25 (2.64–10.41)
Multiple combination		178	(32.1)		29	(33.0)		1.08 (0.61–1.91)
Other single pesticide class or unknown		176	(31.7)		10	(11.4)		0.38 (0.18–0.80)
Application target								
Soil		31	(5.6)		24	(27.3)		8.50 (4.57–15.79)
Small fruit crops*a*		38	(6.8)		14	(15.9)		4.04 (2.03–8.06)
Leafy vegetable crops*b*		25	(4.5)		8	(9.1)		3.51 (1.49–8.27)
Other*c*		461	(83.1)		42	(47.7)		Reference
Application method								
Aerial application		223	(40.2)		26	(29.5)		0.91 (0.54–1.53)
Chemigation		20	(3.6)		22	(25.0)		8.58 (4.31–17.09)
Other*d*		312	(56.2)		40	(45.5)		Reference
**a**For example, berries, grapes, currants. **b**For example, beets, celery, broccoli, lettuce, spinach. **c**Includes tree fruit or other vegetable crops, other crop categories, landscape and forest, undesired plants, livestock farms, unknown. **d**Includes other ground application equipment, multiple, and unknown.								

*Contributing factors to drift incidents.* Of 299 drift events with information on violations of pesticide regulations, 220 (74%) had one or more violations and accounted for 2,093 cases (89% of cases with violation information; Table 8). However, not all of the observed violations may have directly contributed to the drift exposure. Factors contributing to the drift exposure were identified in 164 events, accounting for 1,544 (52%) cases. Common contributing factors identified for drift events included applicators’ carelessness near or over nontarget sites (e.g., flew over a house, did not turn off a nozzle at the end of the row), unfavorable weather conditions (e.g., high wind speed, temperature inversion), and poor communication between applicators or growers and others. Improper seal of the fumigation site (e.g., tarp tear, early removal of seal), which were identified in nine events, accounted for the largest proportion (60%) of cases with contributing factors identified.

**Table 8:** Violation in and contributing factors to occurrence of drift incidents/exposures.

Drift cases				
Drift events (*n* = 643)		Occupational (*n* = 1,380)		Nonoccupational (*n* = 1,565)	
Variable	*n* (%)		*n* (%)		*n* (%)	
Violation of federal/state pesticide regulation									
Yes		220	(73.6)*a*		971	(85.6)		1,122	(93.2)
No		79	(26.4)		164	(14.4)		82	(6.8)
Unknown/pending		344			245			361	
At least one contributing factor identified*b*		164	(100)		486	(100)		1,058	(100)
Applicator carelessness near nontarget sites*c*		79	(48.2)		49	(10.1)		98	(9.3)
By aerial applicator		56	(34.1)		21	(4.3)		66	(6.2)
Weather (wind, temperature inversion)		75	(45.7)		309	(63.6)		593	(56.0)
Poor/ineffective communication		19	(11.6)		102	(21.0)		11	(1.0)
Improper seal of fumigation site*d*		9	(5.5)		94	(19.3)		837	(79.1)
Inappropriate monitoring*e*		7	(4.3)		118	(24.3)		199	(18.8)
Applicator not properly trained or supervised		5	(3.0)		45	(9.3)		0	(0.0)
Excessive application		4	(2.4)		20	(4.1)		6	(0.6)
Use of inadequate equipment*f*		2	(1.2)		125	(25.7)		2	(0.2)
Other*g*		8	(4.9)		28	(5.8)		206	(19.5)
Distance from application site		NA			700	(100)		728	(100)
≤ 50 feet					66	(9.4)		54	(7.4)
> 50–100 feet					77	(11.0)		29	(4.0)
> 100–300 feet					113	(16.1)		69	(9.5)
> 300 feet–0.25 mile					267	(38.1)		93	(12.8)
> 0.25–0.5 mile					175	(25.0)		256	(35.2)
> 0.5–1 mile*h*					0	(0.0)		116	(15.9)
> 1 mile*i*					2	(0.3)		111	(15.2)
NA, for distance from application site, drift events were not applicable. All percentages for “At least one contributing factor identified“ and “Distance from application site“ were calculated only for cases with available data. **a**The CDPR identified 159 (72%). **b**Cases may have been included in multiple categories. **c**For example, the applicator did not turn off a nozzle at the end of the row, or the crop duster flew overhead. **d**For example, leakage from torn tarp, early removal of seal, or use of contaminated water. **e**For example, did not measure wind speed or did not monitor drift from the application site. **f**For example, used longer spray boom than specified on the label or used sprinklers without required calibration device. **g**For example, treated additional rows without permission, permeable soil type, aerial application with very low height, or building/vehicle ventilator system sucking outside air in. **h**Cases are from three events in California, Louisiana, and Washington. **i**Cases are from two events in California.									

The distance between the application and exposure site was identified in 1,428 (48%) cases (Table 8). Occupational cases accounted for 68% of cases exposed within 0.25 miles of the application site, and nonoccupational cases accounted for 73% of cases exposed > 0.25 miles away.

## Discussion

To our knowledge, this is the first comprehensive report of drift-related pesticide poisoning in the United States. We identified 643 events involving 2,945 illness cases associated with pesticide drift from outdoor agricultural applications during 1998–2006. Pesticide drift included pesticide spray, mist, fume, contaminated dust, volatiles, and odor that moved away from the application site during or after the application. Although the incidence for cases involved in small drift events (< 5 cases) tended to decrease over time, the overall incidence maintained a consistent pattern chiefly driven by large drift events. Large drift events were commonly associated with soil fumigations.

*Occupational exposure.* Occupational pesticide poisoning is estimated at 12–21 per million U.S. workers per year (Calvert et al. 2004; Council of State and Territorial Epidemiologists 2010). Compared with those estimates, our estimated incidence of 2.89 per million worker-years suggests that 14–24% of occupational pesticide poisoning may be attributed to off-target drift from agricultural applications. Our study included pesticide drift from outdoor applications only and excluded workers exposed within the application area. Our findings show that the risk of illness resulting from drift exposure is largely borne by agricultural workers, and the incidence (114.3/million worker-years) was 145 times greater than that for all other workers. Current regulations require agricultural employers to protect workers from exposure to agricultural pesticides, and pesticide product labels instruct applicators to avoid allowing contact with humans directly or through drift (U.S. EPA 2009).

Our study found that the incidence of drift-related pesticide poisoning was higher among female and younger agricultural workers and in western states. These groups were previously found to have a higher incidence of pesticide poisoning (Calvert et al. 2008). It is not known why the incidence is higher among female and younger agricultural workers, but hypotheses include that these groups are at greater risk of exposure, that they are more susceptible to pesticide toxicity, or that they are more likely to report exposure and illness or seek medical attention. However, we did not observe consistent patterns among workers in other occupations. This finding requires further research to identify the explanation. The higher incidence in the western states may suggest that workers in this region are at higher risk of drift exposure; however, it may also have resulted from better case identification in California and Washington states through their higher-staffed surveillance programs, extensive use of workers’ compensation reports in these states, and use of active surveillance for some large drift events in California.

*Nonoccupational exposure.* This study found that more than half of drift-related pesticide poisoning cases resulted from nonoccupational exposures and that 61% of these nonoccupational cases were exposed to fumigants. California data suggest that residents in agriculture-intensive regions have a 69 times higher risk of pesticide poisoning from drift exposure compared with other regions. This may reflect California’s use of active surveillance for some large drift events. Children had the greatest risk among nonoccupational cases. The reasons for this are not known but may be because children have higher pesticide exposures, greater susceptibility to pesticide toxicity, or because concerned parents are more likely to seek medical attention. Recently several organizations submitted a petition to the U.S. EPA asking the agency to evaluate children’s exposure to pesticide drift and adopt interim prohibitions on the use of drift-prone pesticides near homes, schools, and parks (Goldman et al. 2009).

*Contributing factors.* Soil fumigation was a major cause of large drift events, accounting for the largest proportion of cases. Because of the high volatility of fumigants, specific measures are required to prevent emissions after completion of the application. Given the unique drift risks posed by fumigants, U.S. EPA regulates the drift of fumigants separately from nonfumigant pesticides. The U.S. EPA recently adopted new safety requirements for soil fumigants, which took effect in early 2011 and include comprehensive measures designed to reduce the potential for direct fumigant exposures; reduce fumigant emissions; improve planning, training, and communications; and promote early detection and appropriate responses to possible future incidents (U.S. EPA 2010). Requirements for buffer zones are also strengthened. For example, fumigants that generally require a > 300 foot buffer zone are prohibited within 0.25 miles (1,320 feet) of “difficult-to-evacuate” sites (e.g., schools, daycare centers, hospitals). We found that, of the 738 fumigant-related cases with information on distance, 606 (82%) occurred > 0.25 miles from the application site, which suggests that the new buffer zone requirements, independent of other measures to increase safety, may not be sufficient to prevent drift exposure.

This study also shows the need to reinforce compliance with weather-related requirements and drift monitoring activities. Moreover, applicators should be alert and careful, especially when close to nontarget areas such as adjacent fields, houses, and roads. Applicator carelessness contributed to 79 events (48% of 164 events where contributing factors were identified), of which 56 events involved aerial applicators. Aerial application was the most frequent application method found in drift events, accounting for 249 events (39%). Drift hazards from aerial applications have been well documented (CDC 2008; Weppner et al. 2006). Applicators should use all available drift management measures and equipment to reduce drift exposure, including new validated drift reduction technologies as they become available.

*Limitations.* This study requires cautious interpretation especially for variables with missing data on many cases (e.g., age, violation, contributing factors, distance). This study also has several limitations. First, our findings likely underestimate the actual magnitude of drift events and cases because case identification principally relies on passive surveillance systems. Such underreporting might have allowed the totals to be appreciably influenced by a handful of California episodes in which active case finding located relatively large numbers of affected people. Pesticide-related illnesses are underreported because of individuals not seeking medical attention (because of limited access to health care or mild illness), misdiagnosis, and health care provider failure to report cases to public health authorities (Calvert et al. 2008). Data from the National Agricultural Workers Survey suggests that the pesticide poisoning rates for agricultural workers may be an order of magnitude higher than those identified by the SENSOR-Pesticides and PISP programs (Calvert et al. 2008). Second, the incidence of drift cases from agricultural applications may have been underestimated by using crude denominators of total population and employment estimates, which may also include those who are not at risk. On the other hand, the incidence for agricultural workers may have been overestimated if the denominator data undercounted undocumented workers. Third, the data may include false-positive cases because clinical findings of pesticide poisoning are nonspecific and diagnostic tests are not available or rarely performed. Fourth, when we combined data from SENSOR-Pesticides and PISP, some duplication of cases and misclassification of variables may have occurred, although we took steps to identify and resolve discrepancies. Also, SENSOR-Pesticides and PISP may differ in case detection sensitivity because the two programs use slightly different case definitions. Lastly, contributing factor information was not available for 48% of cases, either because an in-depth investigation did not occur or insufficient details were entered into the database. We often based the retrospective coding of contributing factors on limited data, which may have produced some misclassification.

## Conclusion

These study findings suggest that the incidence of acute illness from off-target pesticide drift exposure was relatively low during 1998–2006 and that most cases presented with low-severity illness. However, the rate of poisoning from pesticide drift was 69 times higher for residents in five agriculture-intensive California counties compared with other counties, and the rate of occupationally exposed cases was 145 times greater in agricultural workers than in nonagricultural workers. These poisonings may largely be preventable through proper prevention measures and compliance with pesticide regulations. Aerial applications were the most frequent method associated with drift events, and soil fumigations were a major cause of large drift events. These findings highlight areas where interventions to reduce pesticide drift could be focused.
